# Fathers and HIV: considerations for families

**DOI:** 10.1186/1758-2652-13-S2-S4

**Published:** 2010-06-23

**Authors:** Lorraine Sherr

**Affiliations:** 1 University College London, Research Department of Infection and Population Health, London, UK

## Abstract

**Background:**

Fathers are intricately bound up in all aspects of family life. This review examines fathers in the presence of HIV: from desire for a child, through conception issues, to a summary of the knowledge base on fathers within families affected by HIV.

**Methods:**

A mixed-methods approach is used, given the scarcity of literature. A review is provided on paternal and male factors in relation to the desire for a child, HIV testing in pregnancy, fatherhood and conception, fatherhood and drug use, paternal support and disengagement, fatherhood and men who have sex with men (MSM), and paternal effects on child development in the presence of HIV. Literature-based reviews and systematic review techniques are used to access available data Primary data are reported on the issue of parenting for men who have sex with men.

**Results:**

Men with HIV desire fatherhood. This is established in studies from numerous countries, although fatherhood desires may be lower for HIV-positive men than HIV-negative men. Couples do not always agree, and in some studies, male desires for a child are greater than those of their female partners. Despite reduced fertility, support and services, many proceed to parenting, whether in seroconcordant or serodiscordant relationships. There is growing knowledge about fertility options to reduce transmission risk to uninfected partners and to offspring.

Within the HIV field, there is limited research on fathering and fatherhood desires in a number of difficult-to-reach groups. There are, however, specific considerations for men who have sex with men and those affected by drug use. Conception in the presence of HIV needs to be managed and informed to reduce the risk of infection to partners and children. Further, paternal support plays a role in maternal management.

**Conclusions:**

Strategies to improve HIV testing of fathers are needed. Paternal death has a negative impact on child development and paternal survival is protective. It is important to understand fathers and fathering and to approach childbirth from a family perspective.

## Background

Fathers are intricately bound up in all aspects of family life. Family issues play a part in determining life roles, goals and social environment [1]. Yet within the HIV field, fatherhood is understudied. This is a shortcoming, given that HIV itself is predominantly a sexually transmitted infection, closely intertwined with human reproduction and intimate relationships. As fathers have input over the life course, from conception and birth attendance to child rearing, parenting and grand-parenting, their absence in the literature is stark.

This paper discusses a variety of fathering issues in the presence of HIV infection. Motherhood and parenting are empty concepts if fathers are not consulted, and any sociological or psychological study of families will confirm the central roles that relationships and fathers play. The paper should be read in conjunction with the paper by Hosegood and Madhavan [2] in this *Journal of the International AIDS Society *supplement, which may provide some explanatory pathways for the absence of data on fathers and the underrepresentation of paternal insights and views within the literature.

## Methods

The review focuses on seven topics, which, although not exhaustive, provides a synthesis of the existing literature. This takes the form of systematic reviews when there is a sufficient body of data and original empirical data.

The topic, desire for a child, is covered by summarizing the current literature on desire for a child and conducting a specific systematic search on studies looking at childhood desires among men. The coverage was based on a previous systematic review [3] updated and adjusted to focus on men using Pubmed, the Cochrane Database and Psychlit. The search used the key terms, "fertility desire", "pregnancy", "reproductive decision making", "reproductive intentions", "motherhood", "fatherhood", "fathers", "men", "males" and "parenthood". Papers were then restricted to those which included mention of HIV and AIDS.

The topics of HIV testing in pregnancy, fatherhood and conception, fatherhood and drug use, paternal support and disengagement were summarized and reviewed based on a synthesis of published literature. For these topics, the paucity of data rendered a systematic review inappropriate.

The topic, fatherhood and men who have sex with men (MSM), was reviewed based on a synthesis of published literature and supplemented by original data from a survey of male HIV clinic attendees. This information was gathered in the UK [4]. The published data from this study focused on heterosexual, HIV-positive men (n = 32). For the purpose of this report, the data from 84 men who self-reported their sexuality as MSM" were included.

Questionnaire responses were gathered from consecutive male attendees at a London HIV clinic (n = 168) in a study approved by the local ethics committee. One hundred and sixteen men agreed to participate (69.1% response rate). Of these, 84 (72.4%) self-reported their sexuality as MSM signed an informed consent form, were provided with information as to the purpose of the study, assured of anonymity and completed a detailed 17-item questionnaire. The questionnaire included an examination of background demographic issues, parenting experience, attitudes toward parenthood, information needs in relation to reproductive support and service provision, decision making and possibility of unprotected sex, and the meaning of fatherhood.

Questions were rated on Lickert-type or forced choice scales, derived from scales previously utilized in a study of maternal attitudes to parenting in the presence of HIV infection. The data was analysed using SPSS

The section, Fatherhood and child development, was based on the findings from the Joint Learning Initiative on Children and AIDS report [5]. A systematic review of child outcome, orphaning and HIV formed the basis of this report. Data on paternal death were extracted for this article.

## Results

### Desire for a child

For men, fertility, status and lineage considerations all contribute to fertility desires. Most men reside in prenatal societies. Antle *et al *[1] described the importance that parenting has to people living with HIV, who saw it as a joyous part of their lives. In a large US representative sample of HIV-positive males, 28-29% desired children in the future [6].

A systematic review [3] of pregnancy desires identified 29 studies exploring reproductive intentions among HIV positive groups of people internationally. Twenty were studies of women only, seven explored views of men and women, and two examined the views of men exclusively.

Data on men who have sex with men and bisexual men are particularly elusive. Indeed, some studies on parenting desires conducted among HIV populations specifically exclude men who e have sex only with men [6,7]. Men continue to desire fatherhood in the presence of HIV, whether from the United Kingdom [4], South Africa [8,9], Brazil [10,11] or Uganda [12]. In one study in Uganda, men were more likely than women to desire children in the presence of HIV [13]. This was confirmed in a study in Nigeria [14], which also showed a desire for multiple children by men who were newly diagnosed and who had not disclosed their status. A USA study of 2864 people living with HIV showed that 59% of males expected to have a child in the future, but 20% of their female partners were not in agreement [6].

Yet when men and women with HIV are compared to HIV-negative groups, relatively lower fertility desires are reported. A study in Uganda showed a six-fold decrease in desire for children in the presence of HIV [12].

A systematic review of the terms, "pregnancy intentions in HIV" and "males/men", was carried out. The term, "pregnancy intentions", generated 1 122 studies, but when combined with HIV, this reduced to 66. When combined with "men and/or male", 28 studies remained. Hand sorting to meet inclusion criteria (male data, quantitative or qualitative methodology, pregnancy intention outcomes) revealed 14 relevant studies, 10 quantitative and four qualitative. These studies are summarized in Table 1.

**Table 1:** Summary of studies looking at pregnancy and reproduction intentions among men with HIV

Study	Country	N	Gender (sexuality if given)	Fatherhood finding
Paiva *et al *2003 [10]	Brazil	250	Men	43% desired children. Low support and input from health care and reproductive health providers.
Paiva *et al *2007 [11]	Brazil	739	Women (533), men (206): bisexual and heterosexual	Desire for child (27.9%) more frequent among men than among women (50.1% versus 19.2%). Bisexual men more likely to desire biologic children. Male gender, younger age, having no children, living with 1-2 children, and being in a heterosexual partnership were independently associated with desire to have children.
Heys *et al *2010 [12]	Uganda	421 (199 HIV+, 222 HIV-)	Men (36%) and women (64%): heterosexual	Odds ratio of wanting to stop childbearing was 6.25 times greater (p < 0.01) for people living with HIV.
Sherr & Barry 2004 [4]	UK	32	Men (heterosexual)	59% said fatherhood gave meaning to their lives, high fertility desire, low support and access to health care input on reproduction.
Sherr & Barry 2003 [66]	UK	84	Men (MSM)	77.6% said there was no discussion with a doctor about becoming a parent. 68.2% felt they were not sufficiently informed.
Chen *et al *2001 [6]	USA	1421	Men and women ** (M = bisexual or heterosexual, F = heterosexual)	59% of men expect to have a child. 20% of men who desire child have a partner who does not desire child.
Cooper *et al *2009 [8]	South Africa	459	Men (174) and women (285): heterosexual	57% of men were open to possibility of having children. Intention associated with being male, having fewer children and ART treatment.
Nakayiwa *et al *2006 [13]	Uganda	1092	Men (408) and women (604): heterosexual	Men 4 times more likely to want children than women.
Oladapo *et al *2005 [14]	Nigeria	147	Men (52), women (95): heterosexual	71% of men intended to have a child (or more than one). Non-disclosure of HIV status to partner increased odds of child desire.
Panozzo *et al *2003 [7]	Switzerland	114	Men (68) and women (46) **	38% expressed a desire for children, women more often than men.
Cooper *et al *2007* [89]	South Africa	61	Men and women	Strong reproductive desires; treatment availability enhanced such desires.
Ko and Muecke 2005* [90]	Taiwan	8	Men and women	Optimism and high information seeking.
Smith and Mbakwem 2007* [91]	Nigeria	22	Men and women	Treatment enables reproductive goals for both men and women.
Ndlovu V *et al *2009* [15]	Zimbabwe	15	Couples (men and women, at least 1 HIV+)	Treatment availability transformed intentions. None had extinguished intentions.

* = qualitative studies

** = studies which specifically excluded men who have sex with men exclusively

Table 1 shows consistent reports of a desire for fatherhood, with one qualitative study noting that none had "extinguished" the desire for a child. Despite this desire, many studies report a lack of information, services, advice and support.

### HIV testing in pregnancy

HIV testing in pregnancy has become a standard facility available within pregnancy care. Yet, historically, this has been focused on women, with few attempts to include partners, despite the fact that it is highly cost-effective to offer screening to male partners [16] to record discordancy and to reduce the possibility of transmission of HIV during pregnancy. Late HIV infection during pregnancy may result in undetected HIV, missed opportunities for antiretroviral treatment and an elevated chance of vertical transmission. Policy has been very slow to change, despite the fact that testing women only is counterproductive, enhances stigma and leaves men out of the cycle of medical care [17].

Studies on couples testing show this approach to be viable. It results in reduced stigma, enhanced treatment uptake and reduced risk exposure in the event of discordancy [18-20]. Male partner testing is increased when women attending antenatal clinics are requested to invite their partners to attend [21,22]. Couple counseling has shown greater levels of disclosure and acceptance [21,22]. Women are more willing to accept HIV testing in pregnancy if their partners are tested at the same time [23] or even simply attend the clinic with them [24]. The converse is also true in that fear of negative responses from male partners is a disincentive to women testing [25,26]. Yet randomized, controlled trials show low uptake of couples testing, and innovation is needed to reach out more systematically to men [27].

It is also curious to note that HIV testing has been confined to antenatal care with no parallel provision in family planning [28], termination of pregnancy clinics [29] or well-men (or women) clinics. This probably reflects a focus on the infant and may be short sighted. Provision for men at multiple venues may be a more effective strategy.

### Fatherhood and conception

Conceiving a baby is an issue of heightened concern in the presence of HIV. The advent of new therapies and the growing knowledge base has meant that parenting in the presence of HIV is a realistic option [30,31]. Attainment of fatherhood in the presence of HIV is affected by discordancy and by which partner is HIV positive. Interventions concentrate on reduction of viral load, which in turn reduces (but does not eliminate) risk of infection.

The key considerations relate to the risk of infection at the point of conception, reduced fertility as a result of HIV infection, prevention of infection to the infant, and access to support and services in the process of conception and pregnancy. Fertility care and treatment is well established as an HIV-associated service need. In 2001, 75% of fertility clinics surveyed in the UK had a policy of offering treatment to HIV-positive couples [32].

Reduced fertility and problems in conception have been reported in many couples who wish to have a baby, but who do not want to expose an uninfected partner to HIV [33]. Reduced motility of sperm has been noted in the presence of antiretroviral treatment [34,35]. Treatment access is often limited, with ethical and referral barriers reported. Infertility problems were confirmed in a Spanish study [36] with abnormal semen parameters in 83.4% of HIV-infected and 41.7% of HIV-uninfected partners of 130 HIV-positive women. In an African study, there was a high level of risk exposure for non-infected male partners of HIV-positive women desiring pregnancy [37].

Where the man is HIV positive, conception has been documented in the presence of semen washing and in timed unprotected sex. For HIV-positive men, there are four options [38]. Three remove the possibility of genetic parenting: donor sperm insemination (which reduces the risk of viral transmission), fostering and adoption. Sperm-washing techniques have been well described for a number of years and are based on the finding that HIV does not attach to live sperm [39-44]. The techniques require high-level technological provision and have been well established as effective, with minimal risks of infection to either the infant or the partner [45-47,30].

Strategies for harm reduction for couples with no access to treatment [49] try to limit exposure of the uninfected partner. The risk of transmission from an HIV-positive man to an HIV-negative woman in studies in the West is quoted as 0.1-0.3% per act of intercourse [49-51]. This may be elevated in the presence of coinfections. The risk of transmission from an HIV-positive woman to an uninfected man is somewhat lower.

Antiretroviral treatment may affect semen viral load. A review of 19 studies concluded that undetectable viral load in semen was possible with effective treatment, and was negatively influenced by poor adherence to treatment and the presence of other sexually transmitted infections [52]. Caution is consistently needed as studies have also established definitive viral shedding, even in the presence of full viral suppression [53-55]. A number of studies have attempted to evaluate the risk of infection to partners when conception is attempted. This varies by viral load, condom use outside of the fertility window and treatment status of the HIV-positive partner [56-59].

When the woman is HIV positive and the man is HIV negative, infection of her partner can be avoided by using artificial insemination techniques. When both partners are concordant for HIV, there is no risk of transmission, but there is potential for super infection with a different (and possibly drug-resistant) viral strain. To avoid this, artificial insemination procedures can be considered [38].

### Fatherhood and men who have sex with men

Men who have sex with men (MSM) have traditionally fathered children in a number of ways, including having children in a previous or concurrent heterosexual relationship [60], forming a partnership with a woman [61], and using artificial insemination, semen donation or surrogate arrangements. They also become fathers by adoption and fostering children.

There has been a distinct lack of focus on the value of children for men who have sex with men (MSM) generally and HIV-positive, MSM specifically. A study in six US cities estimates that more than 7% of MSM and at least one-third of lesbians are parents [61]. Those men who do wish to become parents must overcome pressures of societal "norms" regarding who or what makes the best family. This is heightened for MSM, HIV-positive men, who, if they want to become parents, must overcome additional obstacles.

The thoughts and expectations of HIV-positive women have been researched, but those of men have been neglected. Prior to the HIV epidemic, Bigner and Jacobson (1989, 1992) investigated the value of children to MSM and heterosexual fathers. Comparisons between the two groups found that MSM fathers did not differ significantly in their desires to become parents, although their motivations for becoming parents were significantly different [62-64]. This was noted in differences on two motivational measures, namely tradition-continuity-security and social status.

A review of 23 empirical studies from 1978 to 2000 among children of lesbian mothers or MSM fathers (one Belgian/Dutch, one Danish, two British, and 18 North American) showed that the majority (20) reported on offspring of lesbian mothers, and three on MSM fathers. The study included 615 children with a wide age range who were contrasted with 387 controls on a series of measures. Children raised by lesbian mothers or MSM fathers did not systematically differ from other children on any of the seven outcome domains, including emotional functioning, sexual preference, stigma experience, and gender role behaviour [65].

Data on HIV-positive men in London were available for both heterosexual and MSM clinic attendees [4,66]. Of the 84 MSM, 77.6% had not had any discussion with a doctor or nurse about the possibility of becoming a parent, with 68.2% feeling insufficiently informed. Approximately one-third had considered having children. Four had had a child prior to HIV diagnosis. Only 4.7% felt that they were fully informed about the issue, and 77.6% had not had any discussion with healthcare professionals about becoming a parent. Few men (4.7%) had considered sperm washing and no men had undergone sperm washing.

Three men reported fathering as a result of an unplanned pregnancy and four men had been involved in a planned pregnancy. More than half of the men questioned said that they would not have unprotected sex in order to conceive, although 38.2% would consider artificial insemination, 2.9% would definitely consider adoption, and 10.6% would definitely consider fostering. More than 90% believed that they would experience some discrimination. Of the sample, 29.4% believed that a child gave meaning to life, and 60% agreed with the statement that a baby would give them something to live for.

It is clear that significant proportions of HIV-positive MSM want children and would use a variety of routes to having a child if the opportunity was offered to them.

### Fatherhood and drug use

In many countries, HIV infection among heterosexual groups is clustered around drug use as a risk factor. The issues surrounding fatherhood, HIV and drug use may have a direct effect on families. A study comparing drugusing fathers with a matched control group (n = 224) noted that drug use contributed to compromised fathering [67]. These results may reflect a skewed group as the drug-user fathers in this study were recruited from methadone maintenance programmes and may thus reflect a group already motivated to address or control their drug use and in contact with services.

Despite this potential positive bias, there were significant negative effects impacting on economic resources to support family formation, patterns of pair bonding, patterns of procreation and parenting behavior. The opioid-dependent fathers displayed: constricted personal definitions of the fathering role; poorer relationships with biological mothers; less co-residence with their children; lower economic provision; less parenting involvement; lower self-esteem as a father; and lower parenting satisfaction ratings. The researchers point out that such compromised fathering in itself may cause psychological distress to the father, as well as impaired parenting experience to the child [68].

A study of the adolescent children of drug-using, HIV positive fathers (n = 505) found direct associations between paternal distress and adolescent distress [69]. In addition, they described several indirect pathways, such as the link between paternal distress and impaired paternal teaching of coping skills, adolescent substance use, and ultimately, adolescent distress. They also report on a direct link between paternal drug addiction and/or HIV and adolescent distress. These data suggest that both drug use and HIV impact directly on fathers, as well as on their ability to parent their children.

### Fathers and support of HIV-positive mothers

Although an understudied area, fathers are generally involved in pregnancy and their support may be key in a number of outcomes [84]. Studies that overlook paternal involvement run the risk of missing a crucial element in family composition, family dynamics and decision pathways. Male involvement in feeding decisions has been associated with increased ease of uptake of exclusive breastfeeding [70-72].

On the other hand, lack of male involvement or fear of male negative reaction has been clearly associated with lowered uptake or avoidance of HIV prevention and protection measures [73,74]. Although many women fear negative reaction from partners when HIV-positive status is disclosed, studies have often recorded positive responses, such as support and financial assistance [75]. Disclosure patterns may often be culturally affected, and it is important to understand who the most desired disclosure contacts are [76].

### Paternal disengagement

Within the HIV literature, there is a background echo, which may well be part of an ongoing myth, around paternal disengagement. Paternal disengagement is a concept that may need to be challenged in the absence of sound global data. Positive engagement in household life by men was reported in a longitudinal South African study. This positive engagement was often not supported or acknowledged [77].

There is good evidence that involving men and providing for risk reduction, particularly for men, can be effective. A systematic review of interventions for men and boys provided compelling evidence of the efficacy of such interventions, thus showing that interventions do exist and are effective and the barrier is one of reaching out to men rather, than the absence of effective tools to do so [78].

Yet there may be some variations and shifts in traditional roles, responsibilities and responses. HIV positive mothers were interviewed in Uganda to explore paternal involvement, as well as paternal kin support and future placement plans [79]. They found that half had fathers who were already deceased, one-third had fathers who were alive but non-resident with their children, and only 16% were residing with their fathers and being supported by them. Furthermore, contrary to cultural norms, mothers indicated preference for placement with maternal, rather than paternal, kin.

Families with HIV-positive mothers were compared with families with HIV-negative mothers, and found significant increased paternal absence and disengagement in the families with HIV-positive mothers [80]. In the USA, disclosure of HIV status was seen as similar to other diseases, but fathers disclosed later than mothers [81]. This may reflect clinic practice, and fathers being overlooked or excluded, as much as father behaviour. For example, White reported, "There was HIV discordancy in more than one-fifth of the parents' relationships. In over 46% of the relationships, the HIV status of the natural or birth father was not known because he was either untested or unavailable" [82]. Such a situation clearly indicates that including and reaching out to fathers is a specific strategy and may need a more family-oriented clinic approach to overcome such gaps.

Fathering an HIV-positive child can bring with it many stressors. Fathers of 31 HIV-positive children aged six to 18 years showed significantly elevated levels of both parenting and psychological distress compared with standardized norms [83]. These fathers requested a range of services, such as gender-specific support groups, assistance with child discipline, help with disease management, and support for future coping.

### Fatherhood and child development

Research on child development has increasingly emphasized the importance of fathers [84]. The current era has seen a change in father roles, as well as a growth in understanding of such roles [85]. The literature on child development and paternal role in the presence of HIV is found in the "orphan" literature in studies that differentiate the gender of the deceased parent and explore the impact of paternal death on a variety of child outcomes.

Despite the fact that there is scant literature on the impact of HIV-positive fathers while they are alive, when they die, the ramifications are considerable. Although HIV is a key factor accounting for paternal death, there are other causes of mortality, such as violence, war, other illnesses and accidents. For example, more than a quarter of young South Africans reported that they had experienced a parental death [86]. Parental death in the HIV literature has been clouded by the fact that many studies do not differentiate between single parent death or dual parent death [87]. Furthermore, few disaggregate their data according to maternal or paternal death or proceed to analyze it separately. Those that do (n = 17) provide clear insight into the impact of paternal death on child development.

The findings are summarized in Table 2. Father presence exerts a protective factor on a range of child out comes and age at paternal loss is also important. Paternal death can affect economic environment, maternal mood, maternal health, access to treatment [88], access to schooling, migration from base families, and a number of other health and psychological out comes. These findings are complex as the effects of paternal death have implications on maternal health and wellbeing, as well as on child outcomes.

**Table 2:** Effect of paternal death on child outcome

Study	Design	Sample	Father findings
Thurman *et al *2006 South Africa [92]	Comparison of orphan and non-orphan youth (age 14-18)	N = 1694, 31% classified as orphan	Significantly more likely to have engaged in sex compared with non-orphans (49% vs. 39%). After adjusting for socio-demographic variables, orphans were nearly one and half times more likely than non-orphans to have had sex. Among sexually active youth, orphans reported younger age of sexual intercourse, with 23% of orphans having had sex by age 13 or younger compared with 15% of non-orphans.
Beegle *et al *2009 [93]	Compared groups who lost a parent aged <15 and those who did not	N = 718. Longitudinal study 1990-2004	On average, children who lose their mother before the age of 15 suffer a deficit of around 2 cm in final attained height (mean 1.96; 95% CI 0.06-3.77) and 1 year of final attained schooling (mean 1.01; 95% CI 0.39-1.81). This effect was permanent. Father's death is a predictor of lower height and schooling as well.
Vreeman *et al *2008, Kenya [94]	Association between ART adherence and parental death.	1516 0-14 year olds	33% had both parents living when they started ART. 21% father dead, 28% mother dead, and 18% both parents dead. The odds of ART non-adherence increased for children with both parents dead.
Birdthistle *et al *2008, Zimbabwe [95]	Comparison between orphans and non-orphans (half experienced parental death).	839 adolescents	Increased sexual risk (HSV2 positive, HIV positive or ever pregnant) among maternal orphans (aOdds Ratio = 3.6; 95% CI 1.7-7.8), double orphans (aOdds Ratio = 2.4; 95% CI 1.2-4.9), and girls who lost their father before age 12 (aOdds Ratio = 2.1; 95% CI 0.9-4.8) but not later (aOdds Ratio = 0.8; 95% CI 0.3-2.2). Maternal and double orphans likely to initiate sex early, have had multiple partners, and least likely to use a condom at first sex and to have a regular sexual partner.
Hosegood *et al *2007, Malawi Tanzania South Africa [96]	1988-2004 data from 3 DSS surveys	Incidence of orphanhood doubled over time	Increased orphan prevalence in 3 populations. Paternal death substantially higher than maternal death. Pattern of co-residence in non-orphans predictive of orphan pattern. 77% paternal orphans live with mother and 68% maternal orphans live with father.
Ford & Hosegood 2005 South Africa [97]	Effect of parental death on child mobility	39,163 children 0-17	Survival status and residency of both mother and father affected mobility. Fathers' death from AIDS was not significantly different from other causes of death.
Doring *et al *2005 Brazil [98]	1998-2001 AIDS mortality and healthcare registry data, 1131 orphans identi3 ed, 75.4% participated	Survey data	70% had lost their father and 50% their mother, and 21% had lost both parents. At the time of the survey, 41% of the children lived with the mother, 25% lived with grandparents and 5% lived in institutions. In multivariate analysis, HIV positivity multiplied the child's chances of living in an institution by a factor of 4.6, losing a mother by 5.9, losing both parents by 3.7.
Watts *et al *2005 Zimbabwe [99]	1998-2000 open cohort follow-up data		Paternal orphan incidence (20.2 per 1000 person years) higher than maternal (9.1 per 1000 person years) and maternal orphans lost fathers at a faster rate than paternal orphans lost their mothers. Paternal and maternal orphan incidence increased with age. Incidence of maternal orphanhood and double orphanhood among paternal orphans rose at 20% per annum. More new paternal and double orphans had left their baseline household. Mortality higher in orphans with the highest death rates observed amongst maternal orphans.
Nyamukapa *et al *2005 Zimbabwe [100]	Stratified population survey at 12 sites (1998-2000)		Maternal orphans but not paternal or double orphans have lower primary school completion rates than non-orphans in rural Zimbabwe. Sustained high levels of primary school completion among paternal and double orphans, particularly for girls, result from increased residence in female-headed households and greater access to external resources. Low primary school completion among maternal orphans results from lack of support from fathers and stepmothers and ineligibility for welfare assistance due to residence in higher socio-economic status households.
Crampin *et al *2003 Malawi [101]	1106 off spring of HIV-positive diagnosed adults in 1980s		Death of HIV-positive mothers, but not of HIV-negative mothers or of fathers, was associated with increased child mortality. Among survivors who were still resident in the district, neither maternal HIV status nor orphanhood was associated with stunting, being wasted, or reported ill-health.
Lindblade *et al *2003 Kenya [102]	Compared non-orphaned children under 6 years with those who lost one or both parents	N = 1190	7.9% lost one or both parents (6.4% father, 0.8% mother and 0.7% both parents). No difference between orphans and non-orphans regarding most of the key health indicators (prevalence of fever and malaria parasitaemia, history of illness, haemoglobin levels, height-for-Age z-scores), Weight-for-height z-scores in orphans were almost 0.3 standard deviations lower. This association was more pronounced among paternal orphans and those who had lost a parent more than 1 year ago.
Thorne *et al *1998 European Collaborative Study [103]	Survey study	1123 children born to HIV-infected women, followed prospectively	70% children cared for by their mothers and/or fathers consistently in their first four years of life, by age 8 approximately 60% will have lived away from parents (i.e., with foster or adoptive parents, other relatives or in an institution), irrespective of child HIV status. Maternal injecting drug use, single parenthood and health status were the major reasons necessitating alternative care.
Kang *et al *2008 Zimbabwe [104]	Comparison of orphan versus non-orphan girls	N = 200	Maternal orphans were more likely to be in households headed by themselves or a sibling, to be sexually active, to have had a sexually transmitted infection, to have been pregnant, and to be infected with HIV. Paternal orphans were more likely to have ever been homeless and to be out of school.
Parikh *et al *2007 South Africa [105]	Comparison of orphan and non-orphan children aged 9-16	N = 174; 87 orphans, 87 non-orphans (13 maternal, 30 paternal, 26 double, 19 missing info)	No significant differences in most education, health and labour outcomes. Paternal orphans more likely to be behind in school. Recent mobility positive effect on school outcome.
Timaeus and Boler 2007 South Africa [106]	Household interviews over time waves	5477 reports on children 8-20 years. (approx. 13% maternal orphans, 26% paternal)	Paternal orphanhood and belonging to a different household from one's father resulted in slower progress at school. Absence of father also associated with household poverty (but did not explain falling behind at school).
Bhargava 2005 Ethiopia [107]	Comparison of Maternal AIDS orphan and other orphans	479 maternal AIDS deaths compared with 574 other maternal deaths	The presence of the father in the household did not significantly affect chances of school participation after maternal death. Presence of father in household positive and significant effects on scores on emotional adjustment. If father prepared meals, positive association with mean scores on 60 items of the Minnesota Multiphasic Personality Inventory.
Foster *et al *1995 Zimbabwe [108]	570 households comparison of orphan and non-orphan household	81.8% paternal death, 13.6% maternal, 4.5% double	Paternal family caring in only 16% families.

CI = confidence interval; ART = antiretroviral therapy

## Conclusions

This paper clearly indicates the crucial role of fathers in family life and structure. The piecemeal state of the literature is lamentable. Where there is good evidence, it is clear that fathers and fathering is a central aspect of the HIV epidemic. Fathers play an important role in the family and their assistance can be harnessed if there is sufficient effort. Fathers can support mothers in the difficulties around infant feeding, early weaning and potential HIV disclosure through feeding practices.

Gender studies often explore lack of attention and provision for women, but in terms of family knowledge and response, the HIV literature on men generally and fathers specifically has specific oversights. Within this, some of the marginalized and difficult-to-reach groups are particularly hard hit. Fatherhood in the presence of HIV infection of the father and drug use in developing and resource-constrained countries, and for MSM, is not fully understood. Yet the loss of a father severely impacts on multiple facets of child development. Fatherhood and paternal contribution to families need to move to centre stage.

## Competing interests

The author declares that she has no competing interests.

## Authors' contributions

This manuscript was conceived, drafted and authored by LS. Assistance in gathering references for the systematic review is acknowledged (N. Jahn). Assistance in the empirical study (N. Barry) is acknowledged.
